# C-Fos Regulation by the MAPK and PKC Pathways in Intervertebral Disc Cells

**DOI:** 10.1371/journal.pone.0073210

**Published:** 2013-09-02

**Authors:** Katsuya Yokoyama, Akihiko Hiyama, Fumiyuki Arai, Tadashi Nukaga, Daisuke Sakai, Joji Mochida

**Affiliations:** 1 Department of Orthopaedic Surgery, Surgical Science, Tokai University School of Medicine, Kanagawa, Japan; 2 Research Center for Regenerative Medicine, Tokai University School of Medicine, Kanagawa, Japan; Chang Gung University, Taiwan

## Abstract

**Background:**

The gene encoding c-fos is an important factor in the pathogenesis of joint disease in patients with osteoarthritis. However, it is unknown whether the signal mechanism of c-fos acts in intervertebral disc (IVD) cells. We investigated whether c-fos is activated in relation to mitogen-activated protein kinases (MAPKs) and the protein kinase C (PKC) pathway in nucleus pulposus (NP) cells.

**Methodology/Results:**

Reverse transcription-polymerase chain reaction and western blotting analyses were used to measure the expression of c-fos in rat IVD cells. Transfections were performed to determine the effects of c-fos on target gene activity. The effect of c-fos protein expression was examined in transfection experiments and in a 3- (4,5-dimethylthiazol-2-yl) -2,5-diphenyltetrazolium bromide cell viability assay. Phorbol 12-myristate 13-acetate (*PMA*), the most commonly used phorbol ester, binds to and activates protein kinase C (PKC), causing a wide range of effects in cells and tissues. PMA induced the expression of c-fos gene transcription and protein expression, and led to activation of the MAPK pathways in NP cells. The c-fos promoter was suppressed completely in the presence of the MAPK inhibitor PD98059, an inhibitor of the MEK/ERK kinase cascade, but not in the presence of SKF86002, SB202190, or SP600125. The effects of the PKC pathway on the transcriptional activity of the c-fos were evaluated. PKCγ and PKCδ suppressed the promoter activity of c-fos. Treatment with c-fos inhibited aggrecan and Col2 promoter activities and the expression of these genes in NP cells.

**Conclusions:**

This study demonstrated, for the first time, that the MAPK and PKC pathways had opposing effects on the regulation of c-fos in NP cells. Thus, the expression of c-fos can be suppressed in the extracellular matrix of NP cells.

## Introduction

Low back pain affects up to 85% of people at some point during their lives, resulting in healthcare and related costs in the United States of $100 billion every year [1]. Intervertebral disc (IVD) degeneration is considered to be one of the major causes of low back pain, and *regenerative therapy has been reported fo*r *IVD degeneration* [2- 4]. However, the precise mechanism of lumbar IVD degeneration remains unknown. Therefore, the *molecular mechanisms* involved in *this must be identified*.

Degeneration of the IVD appears to be mediated by several pathways. Among these, the mitogen-activated protein kinases (MAPKs) in chondrocytes and the IVD have received most attention recently, although little is known about their expression and activity in relation to the progression of IVD degeneration. Three major MAPKs, including extracellular signal-regulated kinase (ERK), c-Jun N-terminal kinase (JNK), and p38 kinase, are regulated by distinct signal pathways that control many aspects of cellular physiology, including cell growth, differentiation, and apoptosis [5-7]. Activated MAPKs regulate gene expression through phosphorylation of downstream transcription factors. Activator protein-1 (AP-1) is a downstream transcription factor in the MAPK pathways that interacts with regulatory DNA sequences known as 12-*O*-tetradecanoylphorbol-13-acetate response elements or AP-1 sites [8]. AP-1 transcription factors are homo- or heterodimers of members of the Jun, Fos, ATF, and Maf families of proteins, all of which are bZIP proteins. AP-1 induction has numerous downstream effects on many cellular processes including proliferation, differentiation, and apoptosis [9-12]. Among the AP-1 complexes, c-fos is a transcription factor that inhibits chondrocyte differentiation [13]. Tsuji et al. [14] reported that introduction of exogenous c-fos into chondrocytes decreases the endogenous transcription of proteoglycans and the tissue inhibitor of metalloproteinase-1, and increases the transcription of matrix metalloproteinase (MMP) -3. Therefore, the c-fos gene plays an important role in both cartilage homeostasis and in the pathogenesis of cartilage destruction.

The activity of c-fos is modulated by interactions with other transcriptional regulators and is controlled by upstream kinases. Some groups have reported that the expression and activation of c-fos are regulated by MAPK pathways in various cell types [15,16]. Protein kinase C (PKC) also stimulates the expression of the c-fos gene [17]. The PKC family comprises at least 12 isozymes classified into three subfamilies: the classical, novel, and atypical PKCs (cPKC, nPKC, and aPKC, respectively) [18]. PKC is a serine/threonine kinase that mediates the effects of a large number of hormones, growth factors, and cytokines. PKC is thus considered a key factor in the regulation of cellular proliferation and differentiation [19,20].

Our previous study suggested that activation of PKC by the activator phorbol 12-myristate 13-acetate (PMA) might lead to an increase in matrix synthesis and cell proliferation, thereby inhibiting IVD degeneration [21]. We also showed that, among the AP-1 complexes, c-fos is suppressed by various signals, but its inhibitory action and role in IVDs have not been clarified. In the present study, we performed functional molecular analyses to investigate how c-fos is regulated upstream of the PKC–MAPK pathways, and to evaluate its involvement in IVD degeneration.

## Materials and Methods

### Ethics Statement

Animal experiments were performed according to a protocol approved by the Animal Experimentation Committee of the University of Tokai (Permit Number: 113005), Tokyo, Japan. Rats used for recovering IVD cells were euthanized by injection of an excess amount (100 mg/kg) of pentobarbital sodium (Nembutal, Abbott. Laboratories, North. Chicago, IL), and all efforts were made to minimize suffering.

### Reagents and Plasmids

Plasmids were provided by Dr. Michael C. Naski (University of Texas Health Science Center at San Antonio, San Antonio, TX). The plasmids were aggrecan-Luc (Agg-luc) and collagen type II-Luc (Col2-luc) [22]. We purchased pJC6-Gl3 (c-jun-luc; #11979), pFOS-GL3 (c-fos-luc; #11983), wild-type (WT) PKC-gamma (WT-PKCγ; #21236), WT-PKC-delta (WT-PKCδ; #16386), WT pcDNA3-FLAG-Fos (WT-c-fos; #8966), and CMV500 A-FOS (DN-c-fos; #33353) from Addgene (Cambridge, MA). An AP-1 reporter assay was performed to examine the effects of PKC signaling using the AP1 Reporter Assay Kit (SABiosciences, Qiagen, Valencia, CA). The empty vector pGL4.74 (Promega, Madison, WI) containing the *Renilla* *reniformis* luciferase genes was used as an internal transfection control. *PMA*, the most commonly used phorbol ester [23], binds to and activates *PKC*, causing a wide range of effects in cells and tissues. Calphostin (CalC) is a highly specific PKC inhibitor that interacts with the regulatory domain of the enzyme and inhibits phorbol ester binding at high concentrations [24]. We obtained CalC from Calbiochem (San Diego, CA). The ERK inhibitor (PD98059) and p38-MAPK inhibitor (SB202190) were obtained from Cell Signaling Technology (Danvers, MA). The p38-MAPK inhibitor (SKF86002) and JNK inhibitor (SP600125) were obtained from Sigma-Aldrich (St. Louis, MO).

### Isolation of IVD Cells

IVD cells were isolated from the lumbar discs of 11-week-old Sprague Dawley rats (n = 32) using methods reported by Hiyama et al. [25]. Briefly, the spinal columns were removed under aseptic conditions, and lumbar IVDs were separated. The gel-like nucleus pulposus (NP) was separated from the annulus fibrosus (AF) using a dissecting microscope. The NP tissue obtained was digested for 30 min in a mixture of 0.4% Pronase (Kaken Kagaku, Tokyo, Japan) and 0.125% collagenase P (Boehringer Mannheim, Roche Diagnostics Corp, Indianapolis, IN). The AF tissue was digested for 1 h with 0.4% pronase and then for 3 h with 0.25% collagenase P. This is an enzyme mixture used for the disaggregation of tissues and isolation of cells. The digested tissue was passed through a cell strainer (BD Falcon, Franklin Lakes, NJ) with a pore size of 100 μm and was washed twice with phosphate buffered saline (PBS; GIBCO, Grand Island, NY). The isolated cells were maintained in Dulbecco’s modified Eagle’s medium (DMEM; GIBCO) and 10% fetal bovine serum (FBS; GIBCO) supplemented with 2% (v/v) antibiotics at 37°C in a humidified atmosphere with 95% air and 5% CO_2_. Low-*passage* (*<3*) *cells* cultured in monolayers were used for all experiments (Figure S1) because cells obtained from rat IVD tissues show variable morphology until passages 2-3 [25]. The cultured cells in the monolayer were analyzed with transfection assays, immunofluorescence staining, a cell proliferation assay, and protein and mRNA expression studies.

### Culture of AP-1 Reporter Cells

A stable AP-1 reporter cell line derived from human 293T embryonic kidney cells transfected with a luciferase reporter construct containing three AP-1 binding sites in the promoter (293T/AP-1-luc, Panomics, Inc., Redwood City, CA) was grown in a humidified atmosphere at 37°C under 5% CO_2_/95% air.

### Measurement of the PMA-induced Activation of AP-1

The 293T/AP-1-luc cells were plated into 24-well cell culture plates (Costar, Cambridge, MA) in the above conditions 1 day before treatment. The cells were at about 60% confluence the following day and were fed fresh medium with or without PMA. The cells were placed again under a humidified atmosphere at 37°C under 5% CO_2_/95% air for 24 h. The plate wells were washed gently with PBS (pH 7.4) and lysed with 5 × Passive Lysis Buffer (Promega). The subsequent lysates were analyzed with the Dual-Luciferase™ reporter assay system (Promega) on a TD-20/20 luminometer (Turner Designs, Sunnyvale, CA). The results were normalized for transfection efficiency and are expressed as the ratio of luciferase to pGL4.74 activities (*Renilla* luciferase activity).

### Immunofluorescence Staining

The NP cells were plated in flat-bottom 96-well plates (3 × 10^3^ cells/well) and incubated for 24 h. The cells were treated with reagents, fixed for 10 min with 4% paraformaldehyde, permeabilized with 0.5% Triton X-100(v/v) in PBS for 8 min, blocked with PBS containing 10% FBS for 1 h, and incubated overnight at 4°C with antibodies against c-fos (1:100 dilution; Santa Cruz Biotechnology, Santa Cruz, CA), and aggrecan (1:100 dilution; Acris Antibodies GmbH, Herford, Germany). The cells were washed and incubated with anti-rabbit Alexa Fluor 488 secondary (green) antibodies (Invitrogen) at a dilution of 1:200 and 10 μM 4′, 6-diamidino-2-phenylindole (DAPI) for 1 h at room temperature for nuclear staining. Fluorescence microscopy was used to observe the samples.

### Immunohistochemistry

To gain insight into the expression of c-fos in the IVD and to assess whether there were changes during development, we evaluated the IVD of embryonic mice and postnatal rats. We chose to use embryonic mouse tissue, because the spinal anatomy, cellular composition, and matrix composition are very similar to those in the rat. Freshly isolated spines from rats (3- and 11-week-old) and day 14.5 embryonic day (E14.5) mice were fixed in 4% paraformaldehyde in PBS and then embedded in paraffin wax. At E14.5, the notochord has virtually disappeared from the vertebral bodies, persisting solely in the locations of the future NP cells. Sagittal sections were deparaffinized in xylene, rehydrated through a graded ethanol series, and stained with hematoxylin. Sections were incubated with an anti-c-fos antibody (Cell Signaling) in 2% bovine serum albumin (BSA) in PBS at a dilution of 1:10 at 4°C overnight. The sections were washed thoroughly, and the bound primary antibody was incubated with a biotinylated universal secondary antibody (Vector Laboratories Canada, Burlington, Ontario, Canada) at a dilution of 1:20 for 10 min at room temperature. Sections were incubated with a streptavidin/peroxidase complex for 5 min and washed with PBS, and the color was developed using 3′-3-diaminobenzidine (VECTASTAIN Universal Quick Kit; Vector Laboratories). Negative controls without the first antibody (c-fos) were prepared. Sections of embryonic mice were washed and incubated with anti-rabbit Alexa Fluor 488 secondary (green) antibodies (Invitrogen) at a dilution of 1:200, and with 10 μM DAPI for 1 h at room temperature for nuclear staining.

### 3- (4, 5-Dimethylthiazol-2-yl)-2, 5-diphenyltetrazolium Bromide (MTT) Assay

NP cell proliferation was measured using a modified MTT viability assay, as described [2]. Exponentially grown NP cells were seeded in 24-well plates at 1.5 × 10^4^ cells/well. The cells were treated with recombinant c-fos (1 ng). MTT diluted in serum-free DMEM was added to the culture medium to a final concentration of 0.5 mg/mL. We had no data on the likely protein concentration of c-fos in the NP cells, so the concentration of c-fos protein (1 ng) cited in a previous study was used [26]. The cells were incubated for 2 h at 37°C, the medium was removed, and the precipitated formazan crystals were solubilized in dimethyl sulfoxide. Product formation was measured using a microplate reader (GE Healthcare Life Science, Pharmacia, Stockholm, Sweden).

### Real-time Reverse Transcription-Polymerase Chain Reaction (RT-PCR) Analysis

NP cells were cultured in 10-cm plates (5 × 10^5^ cells/plate) with or without PMA for 24 h, and total RNA was extracted from the cells using the TRIzol RNA isolation protocol (Invitrogen). RNA was treated with RNase-free DNAse I. Total RNA (100 ng) was used as a template for the real-time RT-PCR analyses. The cDNA was synthesized by the reverse transcription of mRNA, as described [21]. Reactions were set up in triplicate in 96-well plates using 1 μL of cDNA with SYBR Green PCR Master Mix (Applied Biosystems, Warrington, UK) to which gene-specific forward and reverse PCR primers for the genes *c-fos*, *ERK1*, *ERK2*, *p38*, *JNK*, *PKCγ*, *PKCδ*, *aggrecan*, and *col2* were added. The primers were synthesized by TaKaRa Bio Inc. (Kyoto, Japan), and are shown in Table 1. PCR reactions were performed in a *7500 Fast* system (*Applied Biosystems*) according to the manufacturer’s instructions. A *control* (housekeeping) gene, for glyceraldehyde 3-phosphate dehydrogenase (*GAPDH*), was used to *normalize* each sample, and the arbitrary intensity threshold (C_t_) of amplification was computed. *GAPDH* is one of the most commonly used housekeeping genes used in comparisons of gene expression data, and numerous studies using it on molecular level changes in disc biology have been reported by normalization using GAPDH [27-29]. Expression scores were obtained by the ΔΔC_t_ calculation method. The relative messenger RNA (mRNA) expression of target genes per that for GAPDH was calculated.

**Table 1:** Transcripts and sequences of each primer used in real -time RT-PCR.

**Gene**	**NCBI number**	**Forward Primer, 5'–3'**	**Reverse Primer, 5'–3'**
**C-fos**	**NM_022197**	**GGGACAGCCTTTCCTACTACC**	**GATCTGCGCAAAAGTCCTGT**
**ERK1**	**NM_017347.2**	**CTGGACCAGCTCAACCACATTC**	**ACTGTAGGTAGTTTCGGGCCTTCA**
**ERK2**	**NM_053842.1**	**GGCACCAACCATTGAGCAGA**	**GATCATTGCTGAGGTGCTGTGTC**
**p38**	**NM_031020.2**	**CCGCCTCAGTATGCAGTCCA**	**GCCACATGTGCAAAGGCATC**
**JNK**	**XM_341399.5**	**GCAGCCGTCTCCTTTAGGT**	**CATTGACAGACGGCGAAGA**
**PKC-δ**	**NM_133307.1**	**CAAAGGCCGCTTCGAACTCTAC**	**GGCCATCCTTGTCCAGCATTAC**
**PKC-γ**	**NM_012628.1**	**TGGAGTCCTGCTGTATGAGATGTTG**	**CAGTTTGTTCCATGATGGCTTGA**
**Aggrecan**	**NM_022190.1**	**TCCGCTGGTCTGATGGACAC**	**CCAGATCATCACTACGCAGTCCTC**
**Col2**	**NM_012929.1**	**GAGGGCAACAGCAGGTTCAC**	**TGTGATCGGTACTCGATGATGG**

### Western Blotting Analysis

Proteins were prepared using the CellLytic NuCLEAR extraction kit (Sigma-Aldrich). All of the wash buffers and the final resuspension buffer contained a 1× protease inhibitor cocktail (Roche Diagnostics Corp), NaF (5 mM), and Na _3_VO_4_ (200 μM). Nuclear or total cellular proteins were separated on a sodium dodecyl sulfate polyacrylamide gel and were electrotransferred to nitrocellulose membranes (Bio-Rad, Hercules, CA). The membranes were blocked with 5% BSA/TBST (50 mM Tris, pH 7.6, 150 mM NaCl, 0.1% Tween-20) and incubated overnight at 4°C in 5% BSA/TBST with anti-c-fos (1:500; Santa Cruz Biotechnology), anti-c-jun (1:500; Santa Cruz Biotechnology), anti-phospho-p44/42 (ERK1/2) MAPK (1:1000; Cell Signaling), anti-p44/42 MAPK (1:1000; Cell Signaling), anti-phospho-p38 (Thr180/Tyr182) MAPK (1:1000; Cell Signaling), or anti-p38 (Thr180/Tyr182) MAPK (1:1000; Cell Signaling). Immunolabeling was detected using an enhanced chemiluminescence reagent (Amersham Biosciences, Roosendaal, The Netherlands). Western blot data were quantified using ImageJ pixel analysis (NIH Image software). β-actin was used as an internal control to confirm equal protein loading.

### Transfections and Dual-Luciferase Assay

IVD cells were transferred to 24-well plates at a density of 3 × 10^4^ cells/well 1 day before transfection. Cells were cotransfected with 100–500 ng of expression plasmids or the backbone vector along with the reporter plasmids. Lipofectamine 2000 (Invitrogen) was used as the transfection reagent. Cells were cultured for 24 h and treated with a specific concentration of PMA. The cells were harvested 24 h after treatment, and a Dual-Luciferase™ reporter assay system (Promega) was used for the sequential measurements of *firefly* and r*enilla* luciferase activities. The results were normalized for transfection efficiency and are expressed as a relative ratio of luciferase to pGL4.74 activities (denoted as relative activity). Relative luciferase activities were expressed by dividing *firefly* luciferase activity with *renilla* luciferase activity for each sample (at least three independent triplicate experiments). NP cells were transfected with a plasmid encoding green fluorescent protein to check the transfection efficiency, which was 60–70% for NP cells. The luciferase activities and relative ratios were quantified using a Turner Designs Luminometer Model TD-20/20 instrument (Promega).

### Statistical Analysis

Data were compiled from at least three independent triplicate experiments, each performed on separate cultures and on separate occasions. The responses are presented as the fold change relative to the untreated control. The data are presented as the mean ± SD. Data were compared between the experimental groups using Student’s *t* test or analysis of variance (ANOVA) for comparison of multiple groups. Significance was accepted at *P* < 0.05 and is denoted with an asterisk in the figures.

## Results

### AP-1 Transcription in IVD Cells

**Figure 1:**
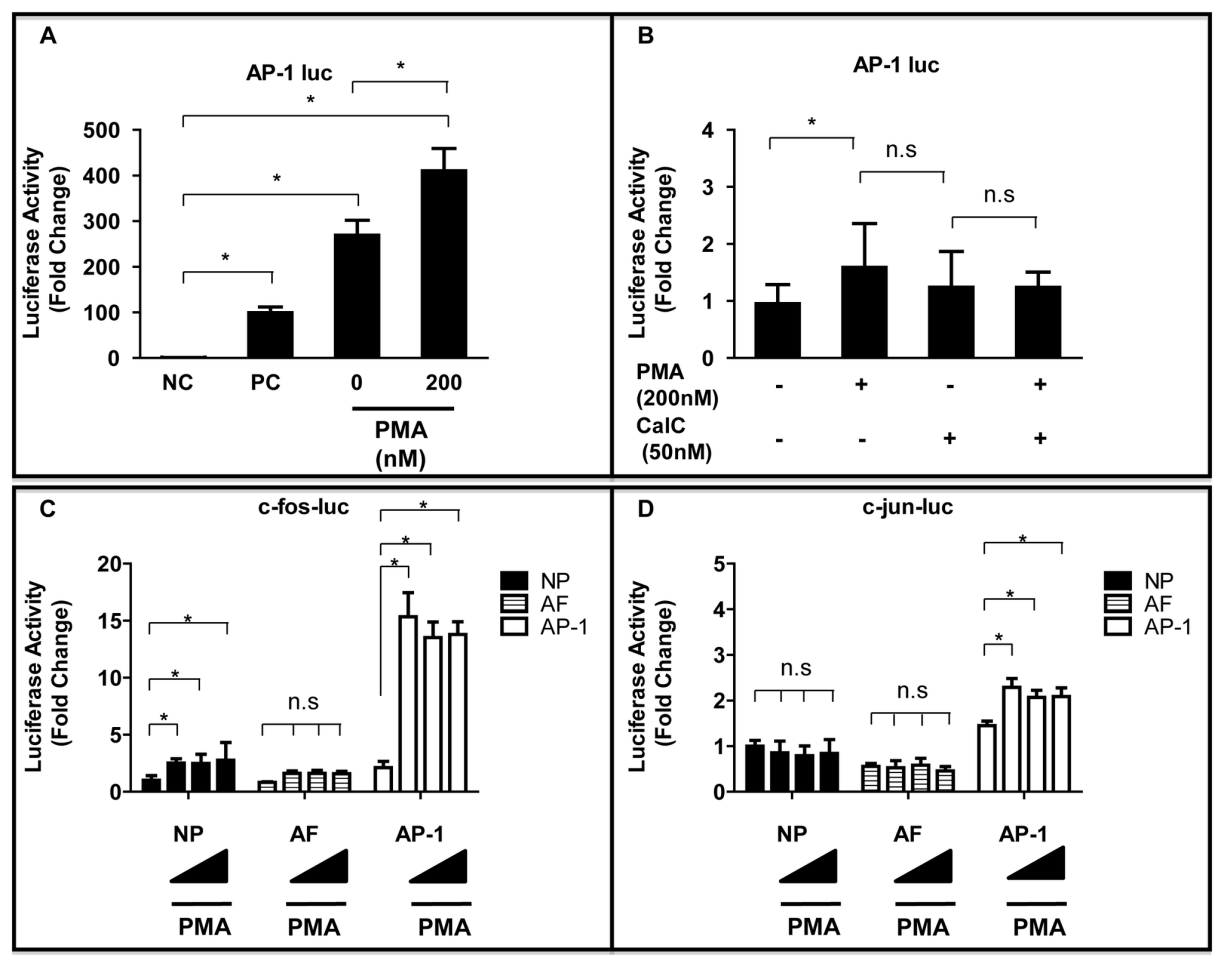
Effect of the PKC activator PMA on AP-1, c-fos or c-jun transcription in IVD cells. (**A**) AP-1 was transfected with the pGL4.74 vector into NP cells, and the cells were stimulated with PMA (200 nM) for 24 h. NC = negative control, (= 1.0 fold); PC = positive control. (**B**) Effect of activation or inhibition of the PKC pathway on AP-1 promoter activity in NP cells. (**C**,**D**) c-fos (**C**) or c-jun (**D**) was transfected with the pGL4.74 vector into NP cells, AF cells, and AP-1 reporter stable cells, and the cells were stimulated with PMA (10, 100 or 200 nM) for 24 h. *P < 0.05 indicates significant differences between groups. Error bars represent the standard deviation (SD). n.s. = not significant.

The transcription factor AP-1 comprises c-fos, c-jun, and related proteins, and it is induced following activation of PKC by PMA. Figure 1A showed that AP-1 promoter activity increased by 1.5 ± 0.8-fold more than the control (null vector) in NP cells after PMA treatment (*P* < 0.05). The fold change in reporter activity was determined by measuring expression in the presence of PMA relative to that of a negative control (set at 1.0). This activity was not suppressed significantly in the presence of the PKC inhibitor CalC (PMA vs PMA + CalC, *P* = 0.245) (Figure 1B). The NP cells, AF cells, and stable AP-1 reporter cells were treated with PMA at the different concentrations indicated to identify the molecular mechanisms underlying PMA activation of the AP-1 complex. Figure 1C and 1D showed that the c-fos promoter activity was activated maximally at 24 h after adding 200 nM PMA in NP cells compared with untreated cells (*P* < 0.05). PMA also stimulated c-fos promoter activity in a dose-dependent manner. Similar findings were observed in stable AP-1 reporter cells transiently transfected with the c-fos construct and treated with PMA for 24 h (*P* < 0.05). However, there were no significant changes in AF cells after addition of PMA compared with untreated cells (*P* = 0.201–0.232). The c-jun promoter activity was also assessed in these cells. It was not induced in NP cells or AF cells in comparison with the respective controls. By contrast, the c-jun promoter activity increased in stable AP-1 reporter cells (*P* < 0.05).

### PMA Enhanced c-fos Transcriptional Activity and Protein Expression in NP Cells

**Figure 2:**
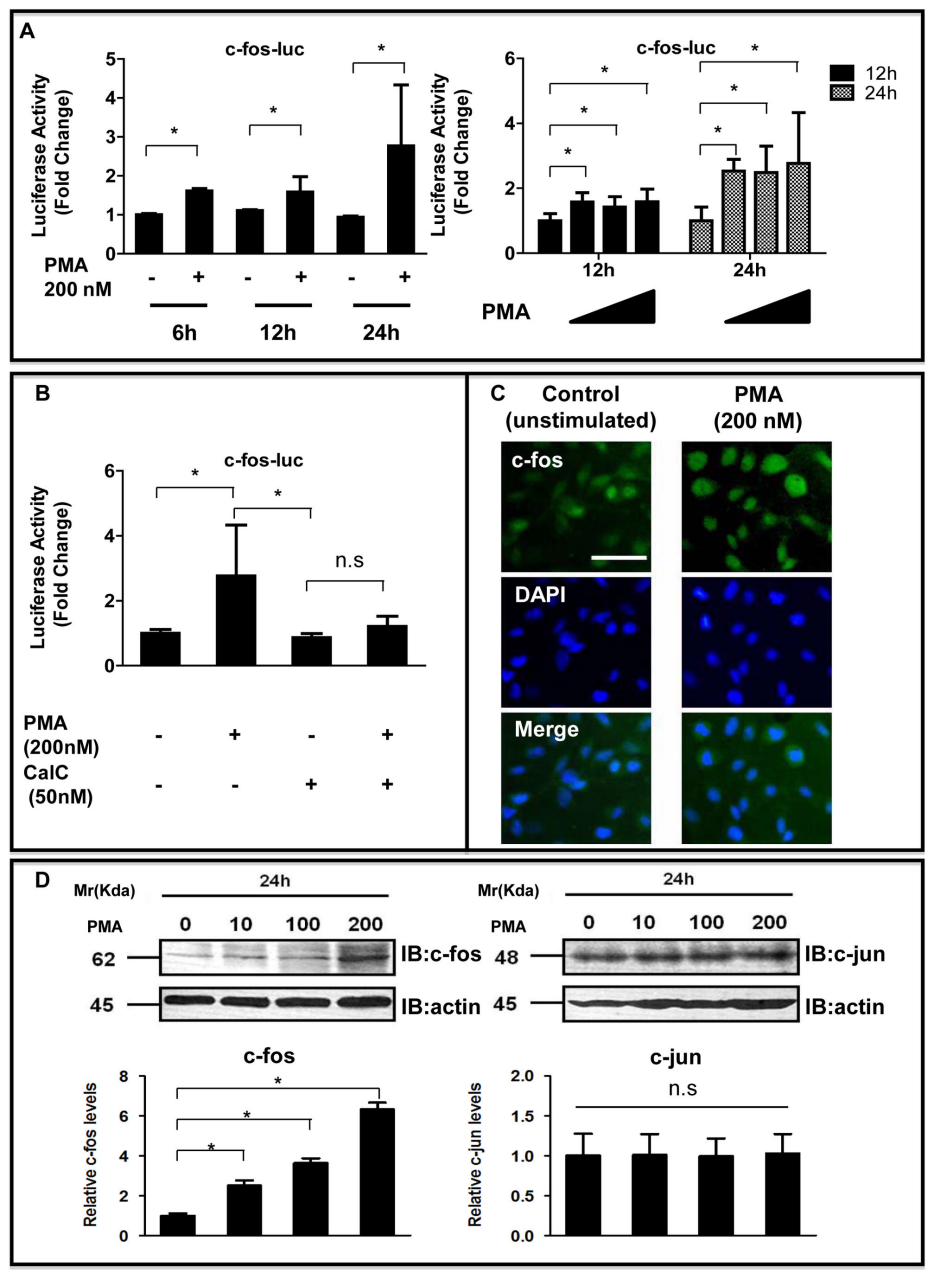
PMA enhanced c-fos expression in NP cells. (**A**) c-fos was transfected with the pGL4.74 vector into NP cells, and the cells were stimulated with PMA (200 nM) for 6–24 h (left panel). In other experiments, c-fos was transfected with the pGL4.74 vector into NP cells, and the cells were stimulated with various concentrations of PMA (0, 10, 100, and 200 nM) for 12–24 h (right panel). (**B**) Effect of activation or inhibition of the PKC pathway on c-fos promoter activity in NP cells. *P < 0.05 between groups. Error bars represent SD. n.s. = not significant. (**C**) Detection of c-fos protein expression by immunofluorescence microscopy. After 1-day of serum deprivation, NP cells were incubated in serum-free medium containing PMA. NP cells were cultured with or without 200 nM PMA for 24 h, fixed, and stained with an antibody against c-fos. **Left**: cells stained with antibody to c-fos. **Middle**: cells stained with DAPI to identify healthy nuclei, (blue). **Right**: cells stained with antibody to c-fos and with DAPI. Scale bar= 50 μm (original magnification 20×). (**D**) Detection of c-fos (left) and c-jun (right) protein by western blot analysis after treatment with PMA (0–200 nM) for 24 h. Densitometry analyses were performed to quantify the levels of c-fos and c-jun protein 24 h after PMA treatment, after normalization to the level of β-actin. *P < 0.05 indicates significant differences between groups.

We next determined whether PMA could induce c-fos transcription and protein expression. Addition of PMA to the culture medium induced c-fos promoter activity in a concentration- and time-dependent manner (*P* < 0.05; Figure 2A). Figure 2B showed that this activity was not suppressed significantly in the presence of the PKC inhibitor CalC (50 nM; *P* = 0.130). Immunofluorescence analysis with an anti-c-fos antibody showed that PMA treatment (200 nM, 24 h) promoted the nuclear translocation of c-fos more strongly in NP cells than in untreated control cells suggesting activation of the c-fos protein (Figure 2C). Western blot analysis with an anti-c-fos antibody also demonstrated that PMA treatment increased the expression of c-fos protein in a dose-dependent manner, but the c-jun protein was not induced in NP cells in comparison with the respective controls (Figure 2D). These data indicated that activation of the PKC pathway stimulated both c-fos promoter and protein expression and confirm that this result was caused by the activation of PKC.

### Expression of c-fos in IVDs

**Figure 3:**
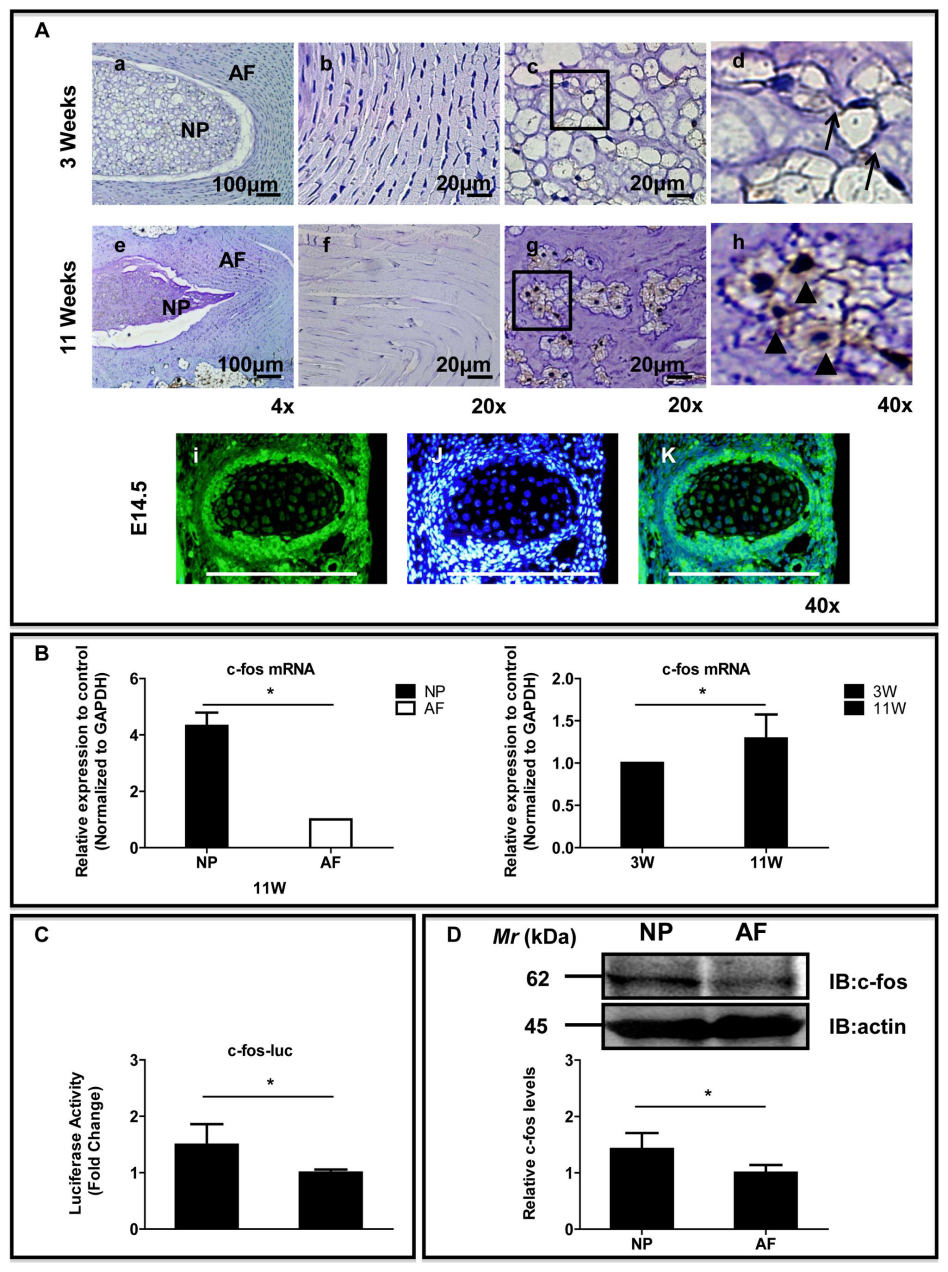
The expression of c-fos in IVD. (**A**) Sagittal sections from 3-week-old (**a**-**d**), 11-week-old (**e**-**h**) rats and a mouse embryo (day 14.5 of gestation). Detection of c-fos expression by immunofluorescence microscopy. **b** and **f**: AF, **c- d** and **g- h**: NP, **i- k**: notchordal cell of embryonic mouse (**Left**: The cells stained with antibody to c-fos. **Middle**: The cells were stained with DAPI to identify healthy nuclei, shown in blue. Right: The cells were stained with an antibody to c-fos and with DAPI). Scale bar= 20 μm-100 μm (original magnification 4×- 40×). (**B**) Real-time RT-PCR analysis of c-fos mRNA levels in NP and AF tissue from 11-week-old rats (left panel); c-fos mRNA levels in NP tissue from 3-week-old rats and 11-week-old rats (right panel). (**C**) Basal activities of the c-fos promoter in NP cells and AF cells were determined by Dual-Luciferase assays. (**D**) Western blot analysis of c-fos expression in AF and NP cells. *P < 0.05 indicates significant differences between groups.

To gain insight into the expression of c-fos in the IVD and to assess whether there were developmental changes, we evaluated the IVDs of embryonic mice and postnatal rats. Sagittal sections of discs from 3-week-old rats, 11-week-old rats and embryonic mice were immunostained with an antibody to c-fos (Figure 3A and Figure S2). We observed weak c-fos protein expression in the NP and AF from 3-week-old rats, but strong c-fos protein expression was observed in both the NP and the AF cells in the 11-week-old rat discs. Expression of c-fos was usually detected in the NP cells. Both cytoplasmic (arrowhead) and nuclear staining of c-fos could be observed in the NP cells from 11-week-old rat discs. In contrast, membranous staining of c-fos (arrow) was observed in NP cells from 3-week-old rats. In addition, it was detected in the notochordal cells from the embryonic mice. We also measured c-fos mRNA expression in tissues to determine any changes in the c-fos gene level in IVDs. The expression of c-fos was significantly higher by 4.32-fold in the NP cells than in the AF cells (*P* < 0.01, Figure 3B). In addition, the gene expression levels of c-fos were slightly higher (1.30-fold) in the NP from 11-week-old rats than in those from 3-week-old rats (*P* < 0.05, Figure 3B). The basal activity of the c-fos promoter was measured in IVD cells. Figure 3C showed that NP cells displayed a significantly higher (1.5-fold) basal level of c-fos promoter activity compared with AF cells (1.0-fold). The expression of c-fos in mature rat cells was studied using western blot analysis. Figure 3D showed that NP cells expressed a 62-kDa band, representing c-fos; expression was more prominent in the NP than in the AF.

### PMA-induced Activation of the MAPK Pathways in NP Cells

**Figure 4:**
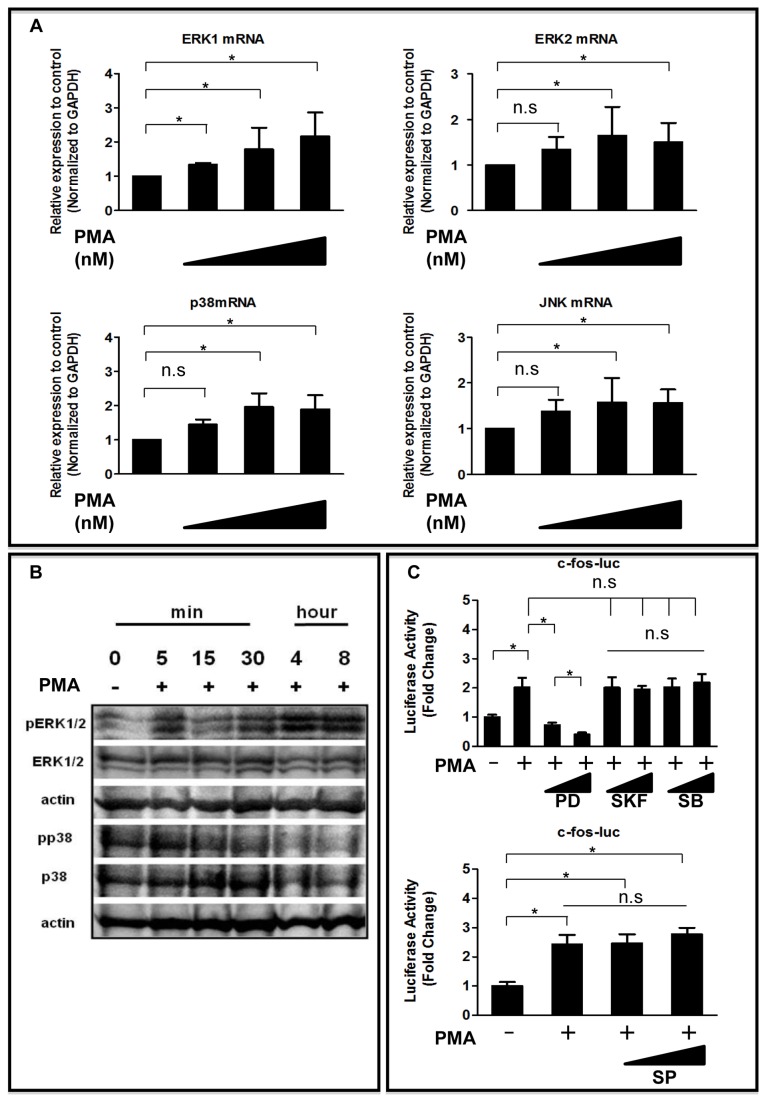
Effect of PMA on MAPK regulation of c-fos expression in NP cells. (**A**) *ERK1*, *ERK2*, *p38*, and *JNK* mRNA expression after exposure of NP cells to PMA (0-200 nM) for 24 h assessed by real-time RT-PCR. (**B**) Western blot analysis of ERK and p38 activation following treatment of NP cells with PMA. After 1-day of serum deprivation, NP cells were treated with vehicle (control) or 200 nM PMA. The same blots were stripped and reprobed with antibodies for total and phosphorylated proteins of ERK or p38. β-actin was detected as a loading control. (**C**) NP cells were transfected with the c-fos reporter plasmid. The transfected cells were then treated with PMA with or without the MAPK inhibitors PD98059 (PD, 30 or 50 μM), SKF86002 (SKF, 5 or 20 μM), SB202190 (SB, 1 or 10 μM), or SP600125 (SP, 1 or 10 μM) for 24 h. *P < 0.05 indicates significant differences between groups; n.s. = not significant.

The role of MAPKs in mediating the PKC pathway was investigated in NP cells. We aimed to identify which of the MAPK pathways was activated by PMA. The expression levels of *ERK1*, *ERK2*, *p38*, and *JNK* mRNA was analyzed by real-time RT-PCR after exposure of the cells to PMA for 24 h. Figure 4A showed that addition of PMA increased *ERK1*, *ERK2*, p38 and *JNK* mRNA levels in NP cells. Figure 4B showed that there was a rapid increase in phospho-ERK (pERK) and phospho-p38 (pp38) levels following PMA treatment. Activation peaked between 5 and 30 min and then declined to basal levels. To identify which MAPK pathways are necessary for PKC-induced activation of transcription factors, the ERK1/2 pathway was selectively blocked with PD98059, the p38 pathway with SKF86002 and SB202190, and the JNK pathway with SP600125. The PMA-induced c-fos promoter activity in the presence of MAPK inhibitors was measured to confirm that PMA-dependent transient activation of the MAPK pathways was required for AP-1 activation. Figure 4C showed that PMA treatment significantly increased the activity of the c-fos promoter; this activity was suppressed completely by the MAPK inhibitor PD98059 but not by SKF86002, SB202190, or SP600125. The activity of the c-fos promoter was measured by performing gain-of-function studies to evaluate further the role of ERK1/2 in transcriptional regulation of c-fos expression. C-fos promoter activity was induced after treatment of NP cells with WT-ERK1. However, no induction of c-fos activity was seen after treatment with WT-ERK2 (data not shown).

### Effects of PKCγ and PKCδ on c-fos Expression in NP Cells

**Figure 5:**
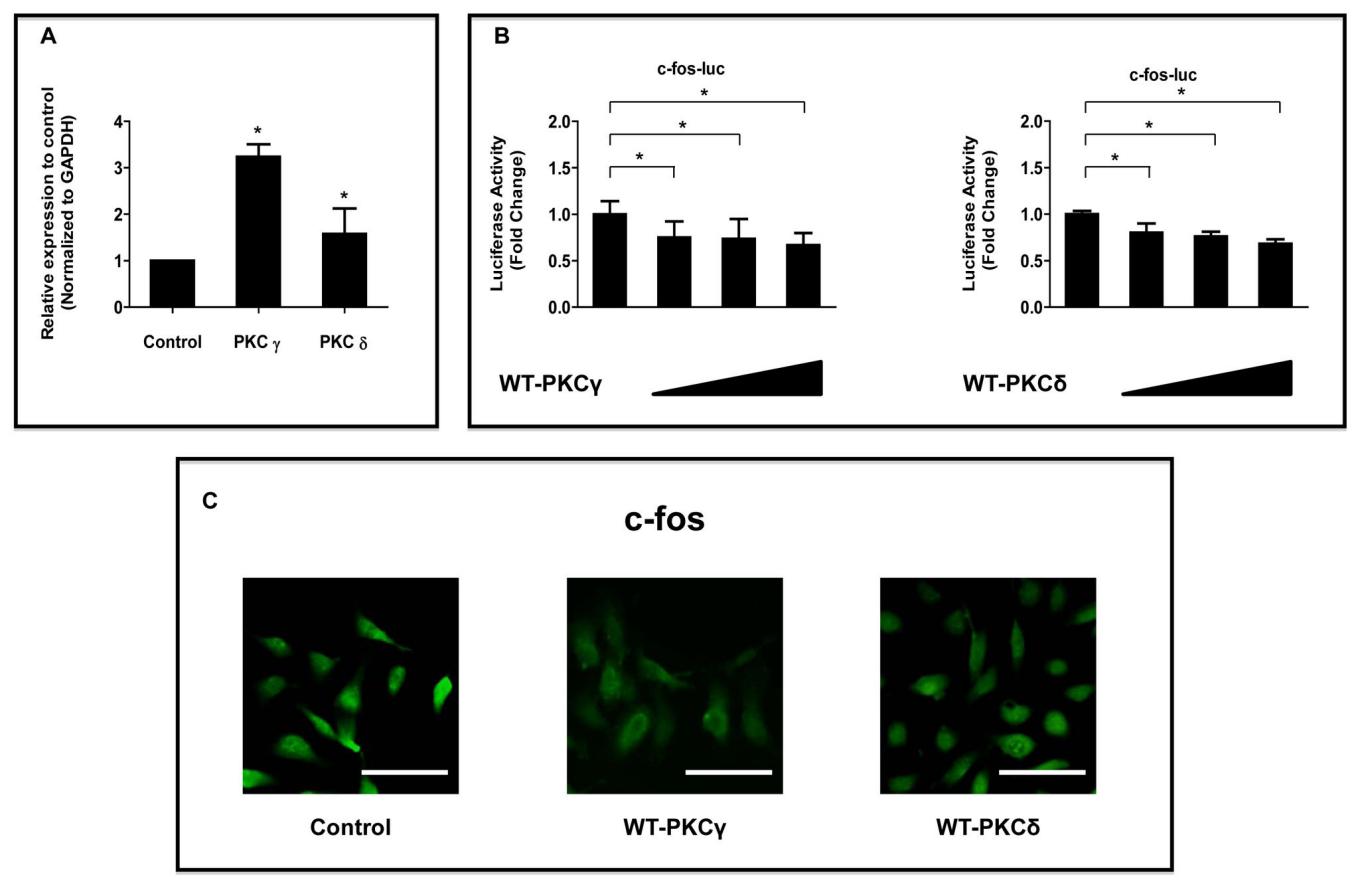
Effect of PMA on PKC regulation of c-fos expression in NP cells. (**A**) Real-time RT-PCR analysis of PKCγ and PKCδ mRNA levels in NP cells treated with PMA (200 nM). (**B**) NP cells were cotransfected with c-fos and increasing concentrations of the WT-PKCγ or WT-PKCδ expression plasmids (100–500 ng). *P < 0.05 indicates significant differences between groups. n.s. = not significant. (**C**) Detection of c-fos protein expression by immunofluorescence microscopy. NP cells were transfected with or without WT- PKCγ or WT-PKCδ expression plasmids, fixed, and stained with an antibody against c-fos (green). Scale bar= 50 μm (original magnification 20×).

*Previous studies demonstrated that PMA* treatment resulted in upregulation of PKC-ε, -γ, and, -ι but not -α or -ζ mRNA. PKC-γ mRNA was highly elevated after PMA treatment. In addition, it has been established that PKCδ is one of the key targets of non-canonical Wnt signaling, in the Wnt/planar cell polarity and Wnt/JNK pathways. Therefore, we investigated the effects of PMA on the expression of the mRNAs of major PKCγ and PKCδ in NP cells in this study [21,30]. Real-time RT-PCR showed that PMA upregulated *PKCγ* and *PKCδ* mRNA expression (200 nM, 24 h) (Figure 5A). To explore further the effect of PKC on the c-fos promoter activity, NP cells were transiently transfected with plasmids encoding the WT form of PKCγ or PKCδ. Figure 5B showed a significant dose-dependent suppression of the c-fos promoter activity in cells cotransfected with WT-PKCγ or WT-PKCδ. Immunofluorescence analysis with an anti-c-fos antibody showed that transfection with WT-PKCγ or WT-PKCδ decreased the expression of c-fos protein in NP cells than in untreated control cells (Figure 5C).

### Role of c-fos Expression in NP Cells Proliferation and Matrix Synthesis

**Figure 6:**
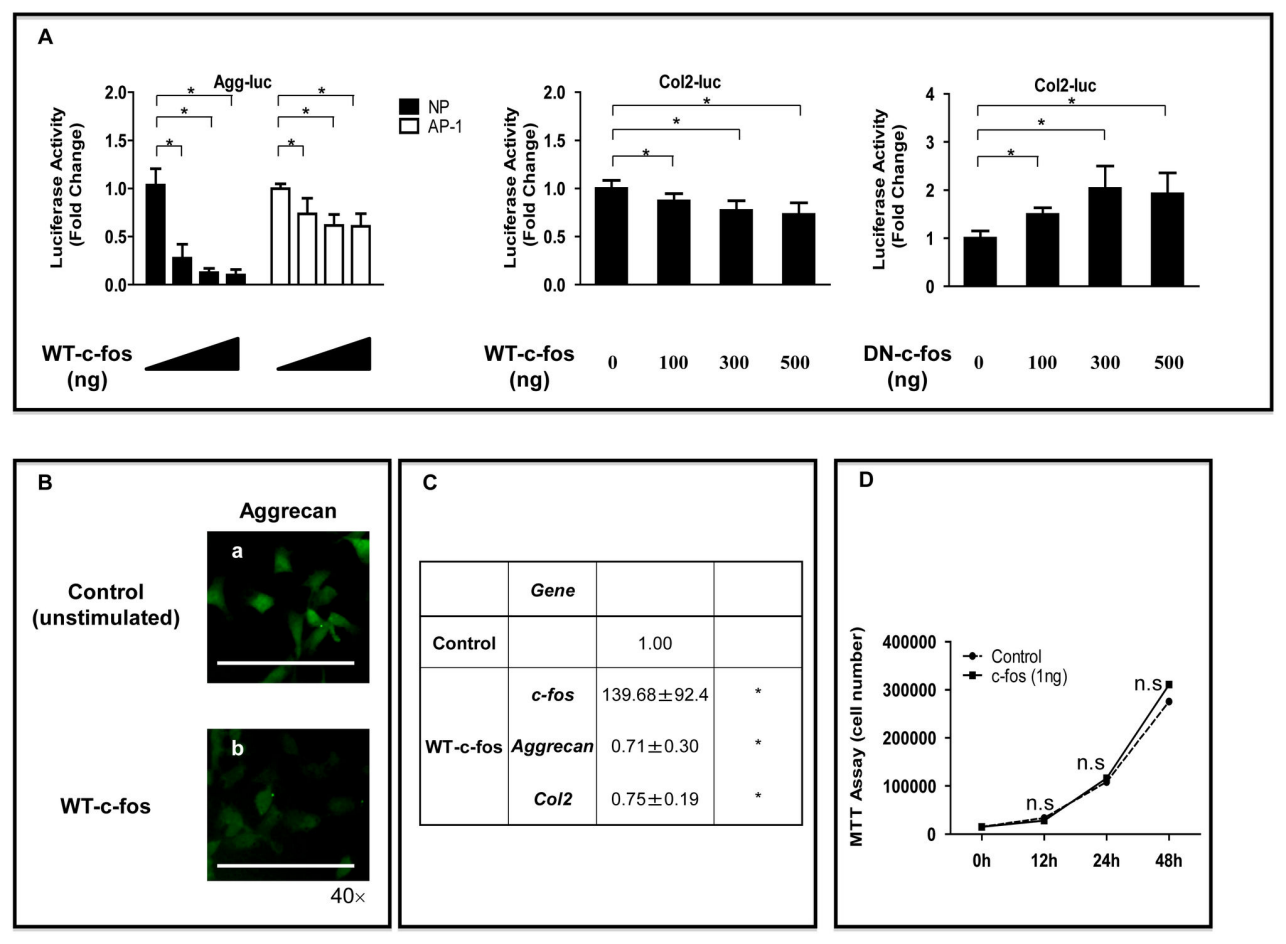
Effect of c-fos on aggrecan (Agg) and collagen type II (Col2) expression and cell proliferation in NP cells. (**A**) NP cells or stable AP-1 reporter cell were cotransfected with the Agg (400 ng) or Col2 (400 ng) reporter constructs and increasing concentrations of the WT-c-fos plasmid (100–500 ng), the DN-c-fos (100–500 ng) plasmid, or empty backbone. *P < 0.05 indicates differences between groups. Error bars represent the SD. (**B**) Detection of Agg protein expression by immunofluorescence microscopy. NP cells were cultured with (**a**) or without (**b**) WT-c-fos for 24 h, fixed, and stained with an antibody against Agg (green). Scale bar= 100 μm(C) Real-time-RT-PCR analysis of c-fos, Agg or Col2 expression in cells treated with vehicle (control) or a WT-c-fos expression plasmid. Total RNA was extracted from transfected cells and real-time RT-PCR was used to determine c-fos, Agg, Col2 and GAPDH RNA levels as an internal control. (**D**) The NP cells were treated with c-fos (1 ng/mL) in 96-well plates. Cell proliferation was evaluated using the MTT viability assay 12 or 24 h after treatment. n.s. = not significant.

To confirm the role of c-fos in controlling the function of the extracellular matrix, the aggrecan and col2 promoter activities and mRNA expression levels were measured in NP cells. Figure 6A showed that the expression of WT-c-fos decreased the aggrecan and col2 promoter activities. The loss-of-function approach was used to confirm data from the gain-of-function experiment. NP cells were cotransfected with the DN-c-fos expression plasmid. There was a dose-dependent increase in aggrecan (data not shown) and col2 promoter activities. Immunofluorescence analysis with an anti-aggrecan antibody showed that treatment with WT-c-fos decreased the expression of aggrecan protein in NP cells more than in untreated control cells (Figure 6B). To address the results obtained from the luciferase reporter assay and immunofluorescence analysis further, we analyzed the mRNA levels of the aggrecan and col2 using the WT-c-fos plasmid by real-time RT-PCR. Figure 6C showed that treatment with WT-c-fos decreased *aggrecan* and *col2* mRNA levels. To further investigate the functional regulation of c-fos in the IVD cells, we next examined the relationship between c-fos and proliferation of NP cells. We found that cell proliferation did not differ significantly between the cells from the control and recombinant c-fos-treated groups (Figure 6D).

## Discussion

Our previous study showed that NP cells induced AP-1 promoter activity following treatment with transforming growth factor-β and bone morphogenetic protein-2 [31]. Another group has shown that PMA treatment activates the MAPK family members ERK1/2 and p38 kinase [32,33]. However, the mechanism responsible for the activation of c-fos is not understood fully. Here we demonstrated that the MAPK pathways were activated by PMA stimulation in NP cells. C-fos activation also increased when the effect of added PMA was suppressed by the ERK inhibitor PD98059. By contrast, c-fos activation was not changed by the p38 inhibitors SKF86002 or SB202190, or by the JNK inhibitor SP600125. These findings suggest that p38 and JNK are not involved in the activation of c-fos by PMA among the MAPK pathways and that regulation of c-fos via ERK might be involved. These findings suggest that c-fos could be activated via the ERK pathway in NP cells, implicating ERK as primary kinase for c-fos transcription in NP cells. Other groups have also shown that IVD cells can be regulated by exogenous and autocrine growth factors through the activation of the MEK–ERK pathway [

].

Previous evidence from *in vivo* studies suggests that elevated c-fos expression affects the differentiation of chondrocytes [39,40]. Genetic studies in mice have provided compelling evidence for the role of AP-1 family members in skeletal development. For example, transgenic mice overexpressing c-fos develop chondro- and osteosarcomatous lesions [41], whereas knockout of c-fos gene expression in mice causes osteopetrosis because of an early block of differentiation in the osteoclast lineage [42]. However, the mechanism underlying the cellular effects of c-fos on the regulation of cell proliferation and the extracellular matrix in IVD cells is not understood.

To date, we have focused on NP cells derived from the IVD because such cells are known to be sparse and have low self-renewal capacity. IVD degeneration is characterized by a number of changes linked to degradation of the extracellular matrix and a decrease in NP cell proliferation [43,44] (Figure S3). Here we measured transcription, gene and protein expression, and cell proliferation in NP cells to ascertain whether c-fos regulates the expression of extracellular matrix proteins such as aggrecan and col2, and cell proliferation. Because the NP is both functionally and embryologically unique, it is important to ascertain whether the regulatory control of aggrecan and col2 mediated by c-fos is specific to this cell type. Stable AP-1 reporter cells were used for this purpose. These showed increased aggrecan promoter activity following treatment with the DN-c-fos plasmid. By contrast, overexpression of c-fos using WT-c-fos directly inhibited the activity and gene expression of aggrecan and col2 in NP cells. The results of these experiments suggest that c-fos is associated with the downregulation of aggrecan and col2 in NP cells. In addition, the MTT as viability assay showed that the number of viable cells did not change significantly up to 24 h after treatment with c-fos. Taken together, these results suggest that although c-fos inhibits extracellular matrix production in the IVD, it is not involved directly in cell proliferation.

The PKC activator PMA increased the expression levels of c-fos and the PKCγ and PKCδ isoforms in NP cells, suggesting that c-fos expression might increase after treatment with these isoforms. However, WT-PKCγ and WT-PKCδ decreased both the transcription and expression of c-fos. These findings suggest: that PKCγ and PKCδ are not major mediators of the PMA-induced increase in c-fos expression, and that other isoforms might be involved directly in the PMA-induced increase in c-fos expression, or that PKCγ and PKCδ downregulate c-fos expression. Amemiya et al. reported that thymeleatoxin, an activator of PKCα, β, γ, increases c-fos gene expression, whereas ingenol, an activator of PKCδ, ε, does not affect the expression in neurons of normotensive rat brains. Further experiments are needed to identify which PKC isoforms increase c-fos expression levels in NP cells.

**Figure 7:**
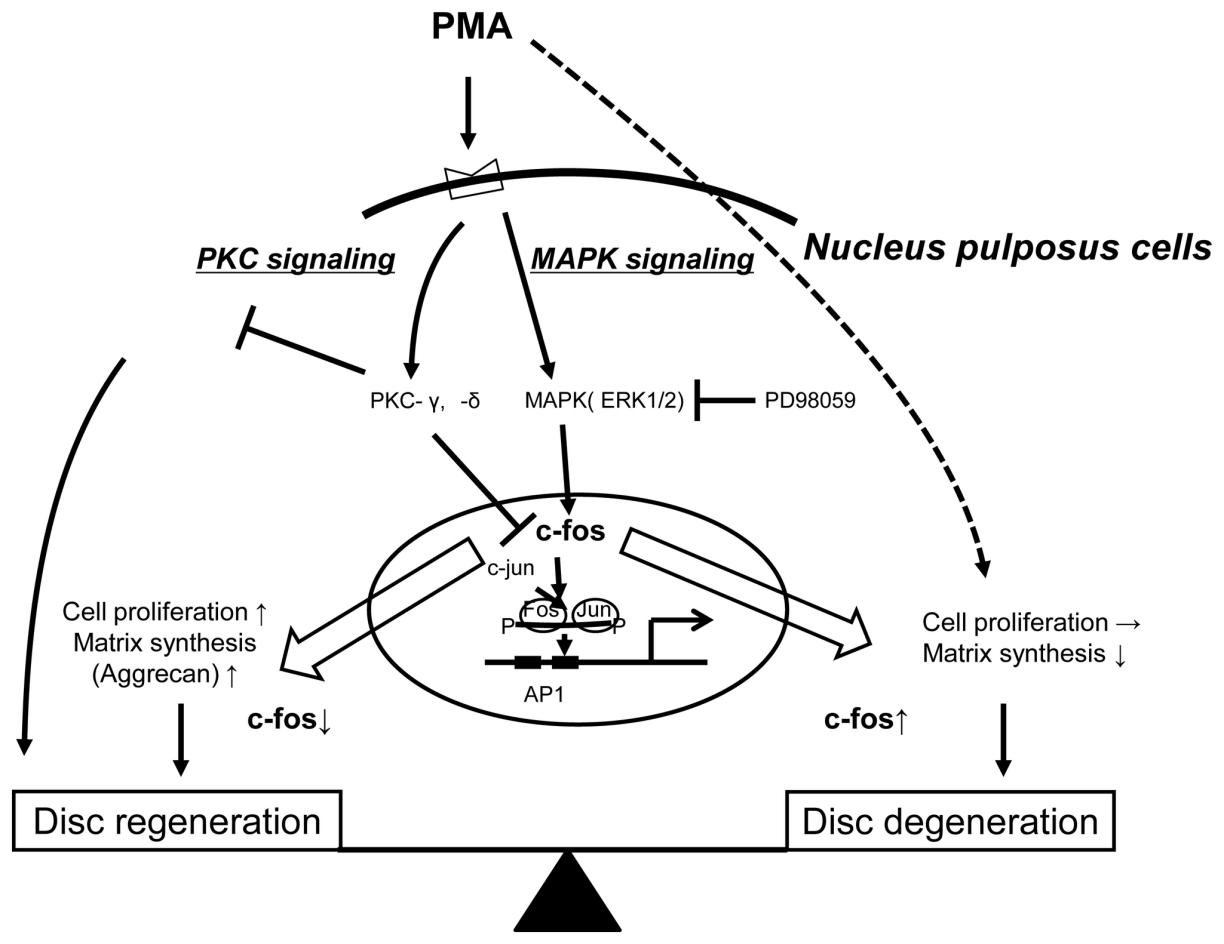
Proposed model for the regulation of c-fos expression by PKC signaling and MAPK signaling in NP cells.

Based on these observations, we propose a model of c-fos regulation by PMA in NP cells that involves both MAPK and PKC pathways (Figure 7). However, this study had a limitation. A previous study found that treatment with PMA induced aggrecan expression in NP cells [21]. However, c-fos promoter activity was induced following treatment with PMA; moreover, c-fos treatment inhibited aggrecan synthesis in NP cells in our study. This was unexpected because the suppression of aggrecan synthesis and proliferation of cells seems to be potentially damaging to the IVD. The reasons for these discrepant findings are not clear, but we suggest three possibilities. First, in this investigation we examined the effect of CalC, a PKC inhibitor, on NP cells with prior exposure to PMA. However, we failed to block the PMA-induced c-fos activation with this treatment. The results demonstrated that part of the stimulatory effect of PMA might not be mediated by PKC. Therefore, the final effect of PMA on aggrecan and col2 gene expression might reflect different effects and factors besides those of c-fos. Second, the different results could reflect cell-specific variations, because we found different results in experiments to measure aggrecan promoter activity between NP and stable AP-1 reporter cells cotransfected with WT-c-fos. Third, these differences might relate to the age and species of the animal from which the cells are isolated and the environment in which the cell metabolism is studied. Future studies for the regulation of c-fos in NP cells from human samples may reveal the molecular mechanisms governing IVD degeneration.

Another important factor to be considered while conducting this study is the potential role of oxygen availability, because NP cells are normally avascular in situ and need distinct support mechanisms to maintain their energy. Therefore, further studies are needed to determine how hypoxia might affect PMA-induced c-fos expression in NP cells.

Inhibition of c-fos by specific pharmacological agents could be useful for reducing aggrecanase-driven IVD degeneration. In particular, ERK1/2-MAPK inhibition significantly inhibited the induction of c-fos expression by PMA. This suggests that blocking ERK1/2-MAPK activity in the NP cells might protect against some of the catabolic actions of c-fos. Our observation that c-fos suppressed extracellular matrix production by IVD cells suggests that the regulation of c-fos expression via MAPK might become a new therapeutic target to prevent IVD degeneration. However, despite the promising potential of MAPK inhibitors, their risks remain of concern. Inhibition of the MAPK pathways could cause general cytotoxicity in NP cells. For instance, inhibition of the p38-MAPK pathway blocks chondrocyte differentiation [45]. Careful screening will be necessary when using MAPK inhibitors to treat IVD degeneration to rule out potential antianabolic or other toxic effects.

In conclusion, these results suggest that PMA treatment increases c-fos gene expression through activation of MAPK pathways and that PKC isoforms might be involved partly in this process [21,25,31].

## Supporting Information

Figure S1 **Morphology of monocultures of NP cells isolated from 11-week-old Sprague Dawley rat lumbar IVDs.** Photomicrograph of primary NP cells cultured in vitro for about 1 week, typical NP cells attached to the culture dish were bright and vacuolar in appearance. Scale bars: 50 μm-100 μm (original magnification 10×- 20×).
(TIF)

Figure S2 **C-fos immunoreactivity in the sagittal sections of IVDs from 3-week-old rats.** Immunohistochemical images of IVD from 3-week-old rats showed moderate levels of c-fos expression and vacuolated morphology. Membranous (arrow) and nuclear (arrowhead) staining can be observed (original magnification 2× or 20×).
(TIF)

Figure S3 **Histology of rat IVDs.** Female Sprangue Dawly (SD) rats (3 and 48 weeks old) were used. Sections of IVDs were stained with hematoxylin and eosin (H＆E) for cell structure and safranin-O (SO) for proteoglycan content. IVDs exhibited a decreased number of notochordal cells with aging, and an age-related decrease in sulfated PGs stained with SO was detected in the NP region of 48 weeks old IVDs compared with the 3 week old specimens. In addition, the older IVD cells were larger and showed more diverse morphotypes the younger ones.
(TIF)
