# Supplementary figures and images for: C-Fos Regulation by the MAPK and PKC Pathways in Intervertebral Disc Cells
Source: PLoS One. 2013 Sep 2;8(9):e73210. doi: 10.1371/journal.pone.0073210 (PMC3759433; doi:10.1371/journal.pone.0073210)

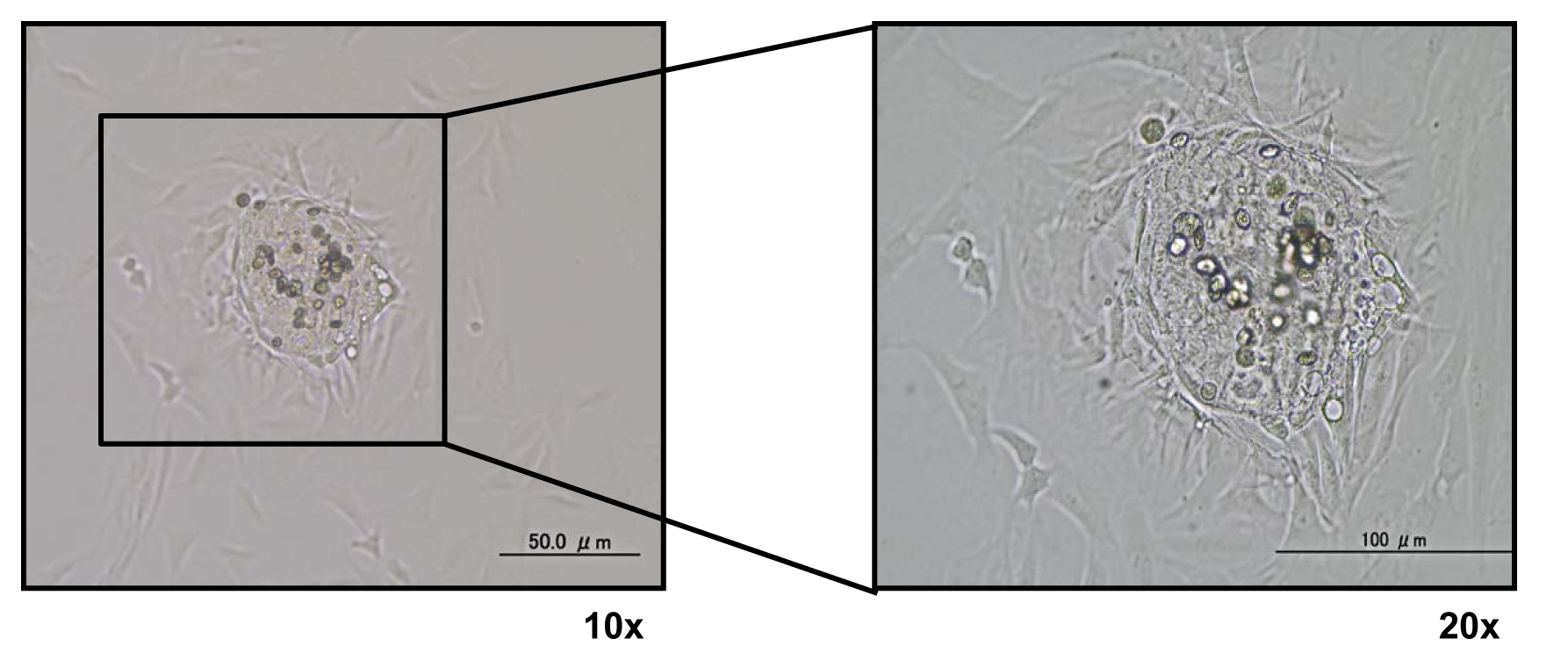

Supplement: Figure S1 — Morphology of monocultures of NP cells isolated from 11-week-old Sprague Dawley rat lumbar IVDs. Photomicrograph of primary NP cells cultured in vitro for about 1 week, typical NP cells attached to the culture dish were bright and vacuolar in appearance. Scale bars: 50 μm-100 μm (original magnification 10×- 20×). (TIF) [file pone.0073210.s001.tif]

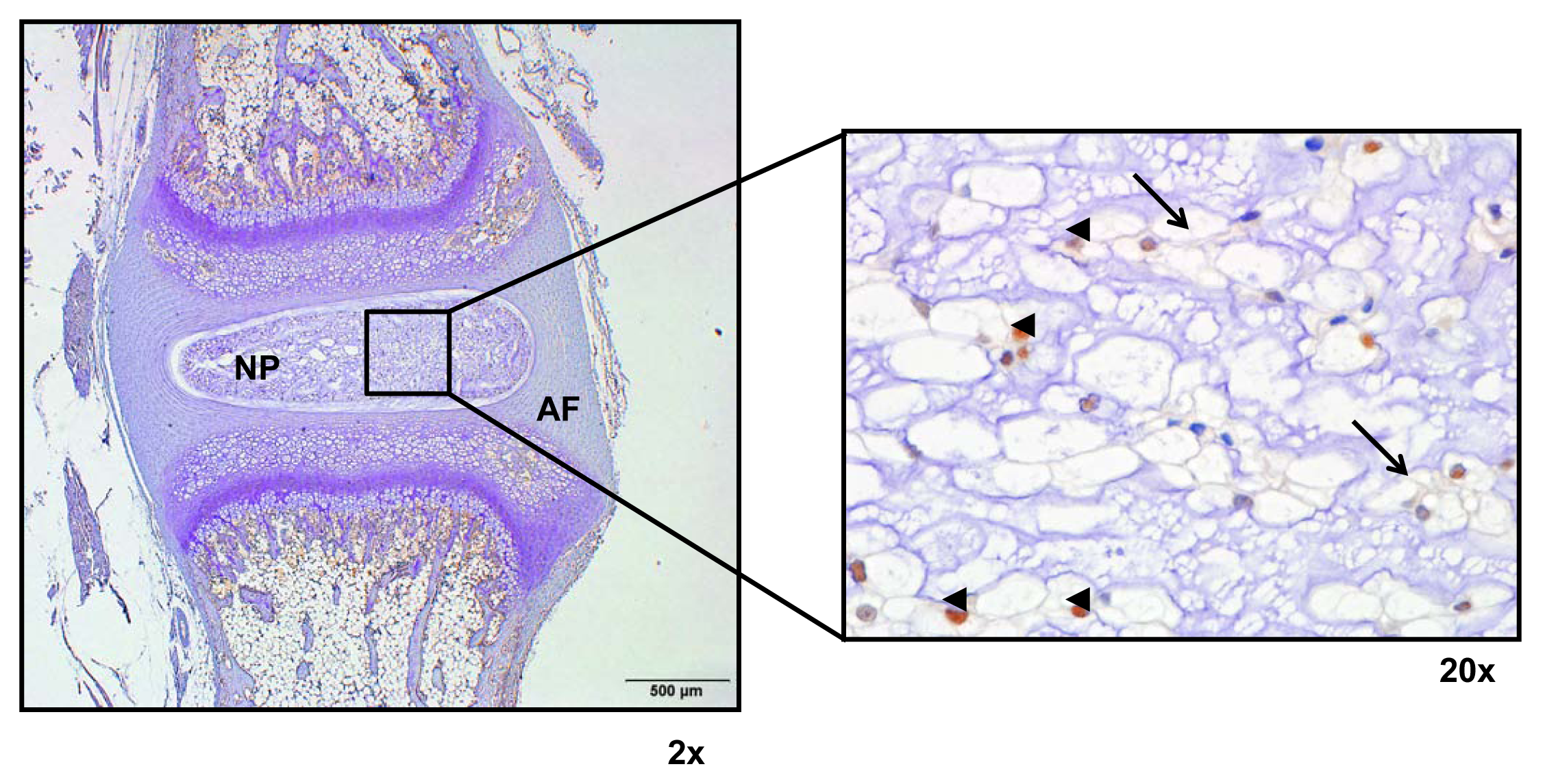

Supplement: Figure S2 — C-fos immunoreactivity in the sagittal sections of IVDs from 3-week-old rats. Immunohistochemical images of IVD from 3-week-old rats showed moderate levels of c-fos expression and vacuolated morphology. Membranous (arrow) and nuclear (arrowhead) staining can be observed (original magnification 2× or 20×). (TIF) [file pone.0073210.s002.tif]

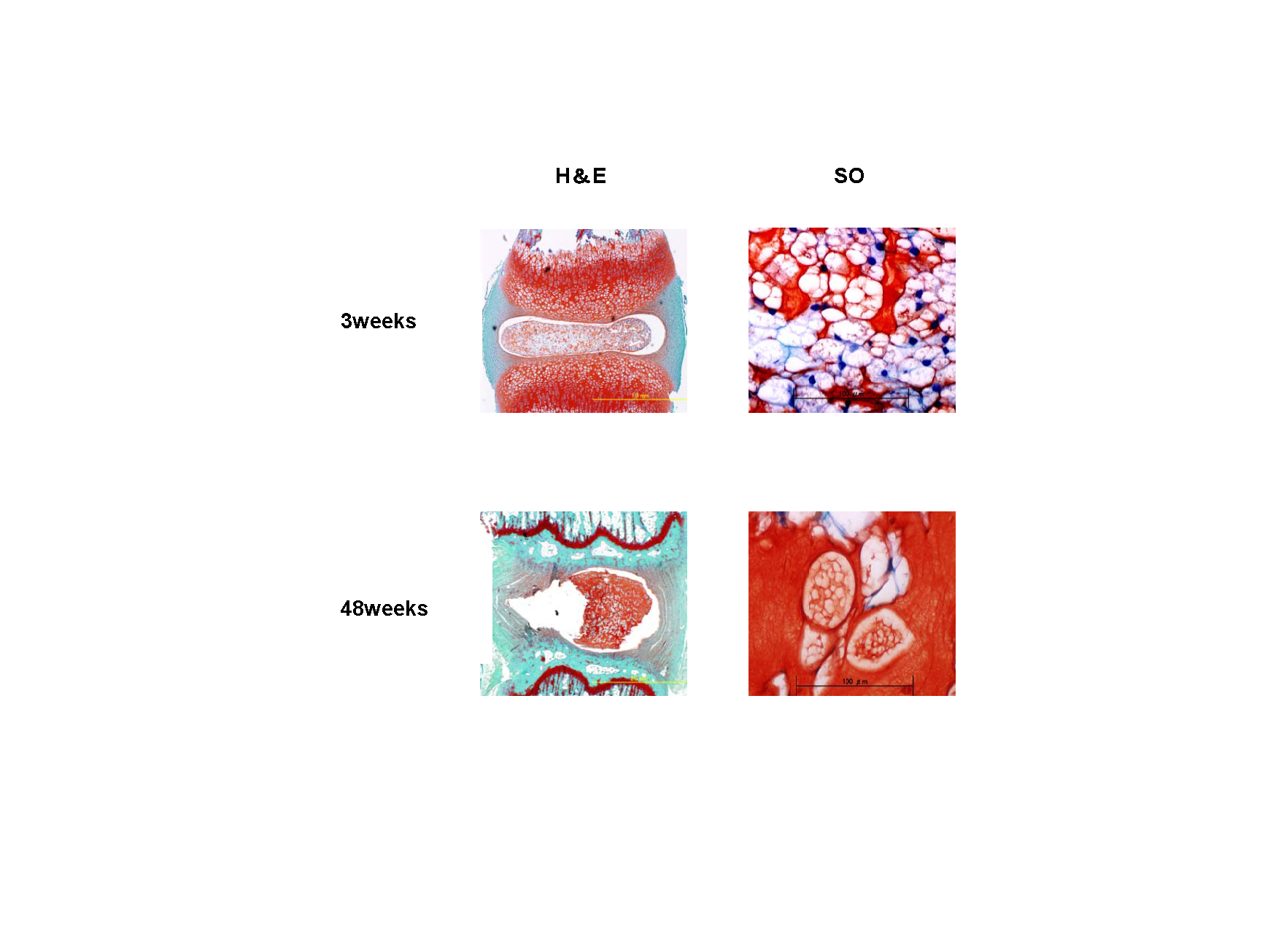

Supplement: Figure S3 — Histology of rat IVDs. Female Sprangue Dawly (SD) rats (3 and 48 weeks old) were used. Sections of IVDs were stained with hematoxylin and eosin (H＆E) for cell structure and safranin-O (SO) for proteoglycan content. IVDs exhibited a decreased number of notochordal cells with aging, and an age-related decrease in sulfated PGs stained with SO was detected in the NP region of 48 weeks old IVDs compared with the 3 week old specimens. In addition, the older IVD cells were larger and showed more diverse morphotypes the younger ones. (TIF) [file pone.0073210.s003.tif]
